# Reconstructing the Periosteal Niche with Regenerated Cellulose Nanofibers for Endogenous Bone Regeneration

**DOI:** 10.3390/jfb17090435

**Published:** 2026-09-01

**Authors:** Siphesihle Cassandra Nonjola, Subin Park, Jeong In Kim, Soonchul Lee

**Affiliations:** 1 Department of Orthopedic Surgery, CHA Bundang Medical Center, CHA University School of Medicine, 335 Pangyo-Ro, Bundang-Gu, Seongnam-Si 13488, Republic of Korea; 2 Department of Anatomy, CHA University School of Medicine, 335 Pangyo-Ro, Bundang-Gu, Seongnam-Si 13488, Republic of Korea; 3 SL Bio, Inc., 43, Beolmal-Ro 30beon-Gil, Bundang-Gu, Seongnam-Si 13503, Republic of Korea

**Keywords:** periosteum-mimetic scaffold, regenerated cellulose, electrospinning, bone regeneration, hydroxyapatite scaffold

## Abstract

Large bone defects remain difficult to treat because current bone substitutes largely restore mechanical integrity without reconstructing the periosteal microenvironment that orchestrates endogenous bone regeneration. Here, we developed a periosteum-inspired composite scaffold by integrating a regenerated cellulose nanofibrous membrane onto a compressed hydroxyapatite scaffold to simultaneously mimic the biological interface and mineralized framework of native bone. A cellulose acetate electrospun membrane was converted into regenerated cellulose through deacetylation while preserving its extracellular matrix-like fibrous architecture, providing a hydrophilic surface favorable for cell–material interactions. The periosteum-mimetic membrane supported cell attachment and increased cellular metabolic activity, demonstrating its ability to establish a regenerative microenvironment at the scaffold surface. In a critical-sized femoral defect model in SD rats, the composite scaffold markedly enhanced bone regeneration compared with the hydroxyapatite scaffold alone, leading to substantially increased newly formed bone area. Histological analysis further revealed elevated expression of the osteogenic transcription factor Osterix, indicating enhanced osteogenic commitment during bone repair. Rather than functioning solely as a structural covering, incorporation of the engineered periosteal membrane provided a cell-supportive interface associated with enhanced bone regeneration. This biomimetic strategy demonstrates that reconstructing periosteal function represents an effective approach for designing next-generation bone grafts with enhanced biological performance and regenerative capacity.

## 1. Introduction

Bone possesses a remarkable intrinsic capacity for regeneration; however, this regenerative potential becomes severely compromised when the defect exceeds the critical size or when the surrounding biological environment is disrupted [1,2,3]. Current bone graft substitutes have therefore focused predominantly on restoring mechanical stability through osteoconductive ceramics such as hydroxyapatite (HAp) or β-tricalcium phosphate [4,5,6,7,8]. Although these materials provide an appropriate mineral framework for bone ingrowth, they insufficiently recreate the complex biological microenvironment that coordinates endogenous skeletal repair [9]. Consequently, limited cellular infiltration, delayed osteogenic activation, and incomplete remodeling remain major obstacles to successful regeneration [4,10,11,12,13].

Among the native skeletal tissues, the periosteum represents one of the most biologically active regenerative compartments [14]. Far beyond functioning as a simple connective tissue covering, the periosteum serves as a dynamic niche composed of an outer fibrous layer rich in extracellular matrix and an inner cambium layer containing osteoprogenitor cells [14,15]. Following injury, the periosteum rapidly orchestrates a cascade of regenerative events by facilitating cell adhesion, directing progenitor cell recruitment, supporting vascular invasion, and regulating osteogenic differentiation through spatiotemporally coordinated biochemical and mechanical cues [16]. Clinical observations consistently demonstrate that preservation of the periosteum markedly improves fracture healing, whereas periosteal damage substantially delays bone regeneration [16,17,18,19]. These findings underscore that successful bone repair depends not only on replacing mineralized tissue but also on reconstructing the biological interface governing tissue regeneration.

Recent advances in bone tissue engineering have increasingly recognized the importance of biomimetic scaffold design [9,20]. Considerable efforts have been devoted to reproducing the hierarchical architecture, stiffness, porosity, and mineral composition of bone [21,22]. However, comparatively little attention has been paid to recreating the periosteal microenvironment, despite its central role in orchestrating skeletal healing. Existing approaches typically rely on growth factor delivery, stem-cell transplantation, or decellularized periosteal grafts, each of which faces limitations including biological variability, manufacturing complexity, limited availability, and regulatory challenges [23]. Accordingly, there remains a substantial need for acellular biomaterials capable of recapitulating key periosteal functions while maintaining structural simplicity and translational feasibility.

Electrospun nanofibers provide an attractive platform for periosteum engineering because their fibrous architecture closely resembles the native extracellular matrix [9,24,25,26]. Among available biomaterials, regenerated cellulose (CEL) exhibits excellent biocompatibility, hydrophilicity, mechanical flexibility, and abundant hydroxyl groups that facilitate protein adsorption and cell attachment [10,27]. Cellulose acetate (CA) can be readily electrospun into uniform nanofibrous membranes and subsequently converted into regenerated CEL through deacetylation without disrupting the fibrous morphology [27,28]. Such structural preservation enables the fabrication of extracellular matrix-like membranes capable of promoting favorable cell–material interactions while avoiding the complexity associated with bioactive factor incorporation [28].

Here, we hypothesized that reconstructing the periosteal interface on the surface of a conventional HAp scaffold would fundamentally improve endogenous bone regeneration by restoring the early biological events that precede mineral deposition. To test this hypothesis, we fabricated a periosteum-inspired composite scaffold by integrating a regenerated CEL nanofibrous membrane onto a compressed HAp substrate. The resulting hybrid scaffold was designed to emulate the complementary functions of native cortical bone and periosteum by combining structural rigidity with an extracellular matrix-mimetic regenerative interface. We systematically investigated its effects on cellular attachment and viability in vitro and evaluated its regenerative efficacy in a critical-sized femoral defect model in Sprague–Dawley rats. Our findings demonstrate that engineering the periosteal microenvironment, rather than modifying the mineral scaffold alone, represents a promising strategy for enhancing bone regeneration through endogenous tissue repair.

## 2. Experimental Section

### 2.1. Materials Preparation

CA (Mn = 30,000; CAS No. 9004-35-7), sodium hydroxide (CAS No. 1310-73-2), acetone (≥99.8%; CAS No. 67-64-1) and N-N-Dimethylformamide (DMF, ≥99.8%; CAS No. 68-12-2) were purchased from Sigma Aldrich (St. Louis, MO, USA). Cellophane tape, copper wire and polycarbonate film were obtained from Cosmotech Inc. (Seoul, Republic of Korea). Polyethylene tubing was purchased from Nano NC (Seoul, Republic of Korea) and 10 mL syringe fitted with a 23-gauge needle from NORM-JECT^®^ (Wolf, Germany). All primary antibodies, unless otherwise specified, were obtained from Abcam (Cambridge, UK) and cell culture reagents were purchased from Hyclone (Logan, UT, USA). Coverslips were purchased from SPL life Sciences (Pocheon, Republic of Korea).

### 2.2. Fabrication of Periosteum-Inspired Composite Scaffolds

Prior to electrospinning, a 16 wt% CA solution was prepared by dissolving CA powder in a DMF/Acetone solvent system mixed in a 1:2 ratio. The solution was stirred for 10 h at room temperature before being loading in a 10 mL syringe.

During electrospinning, the metal spinneret was maintained 15 cm away from the rotating collector spinning at 1000 rpm. The feed rate was maintained at 1 mL/h, and a voltage of 15 kV was applied. Under these conditions, CA nanofibers were deposited onto a polycarbonate film wrapped around the rotating collector. After electrospinning for 8 h, the fiber mats were peeled off from the collector and dried overnight to evaporate residual solvent.

Post-electrospinning, a 0.1 M sodium hydroxide (NaOH) solution was prepared by dissolving sodium hydroxide pellets in distilled water. The composite scaffolds were immersed in the NaOH solution for 30 min, followed by thorough washing with distilled water and drying. All scaffolds were sterilized before use.

### 2.3. Morphological and Physicochemical Characterization of Periosteum-Inspired Composite Scaffolds

Scanning electron microscopy (SEM; JSM-7800F, JEOL, Tokyo, Japan) was used to evaluate the surface morphology of composite CEL scaffolds. An average of 100 randomly selected nanofibers from SEM images were used for fiber diameter analysis via ImageJ Software (Ver. 1.54). The chemical composition of composite scaffolds before and after sodium hydroxide treatment was assessed via Fourier-transform infrared spectroscopy (FT-IR). All spectra were measured in the range of 400 cm^−1^ and 4000 cm^−1^ using a Nicolet iS50 spectrometer (Thermo Fisher, Waltham, MA, USA). The crystalline phases were confirmed via X-ray diffraction (XRD) analysis by collecting diffraction over a 2θ range of 5–60° using an X-ray diffractometer at a scanning rate of 3° per minute. The diffractometer was equipped with a Cu Kɑ radiation source (λ = 1.54Å) and operated at 40 kV and 30 mA.

### 2.4. Mechanical Testing

Scaffolds were prepared in a uniform cylindrical geometry before evaluation of their compressive properties (diameter: 18 mm, height: 10 mm) under dry conditions. Prior to testing using a universal testing machine (Oriental Testing Machine, Oriental Testing M/C, version 12.7.2, Siheung, Republic of Korea), each sample was positioned to ensure proper alignment between the compression platens, and a preload was applied to establish full contact between the specimen and the loading surface. During the test, a constant crosshead speed of 2 mm/min was applied and load–displacement data were recorded continuously. The compressive strength of the scaffolds was calculated based on the maximum load sustained by the specimen prior to failure, normalized to the cross-sectional area.

### 2.5. Cell Morphology Analysis

The morphology of human bone marrow–derived mesenchymal stem cells (hBMSCs) was evaluated to determine the influence of mineral extracts and fibrous scaffolds on cellular behavior. HAp extraction medium was prepared in accordance with ISO 10993-12:2021 by incubating the mineral powder in culture medium at an extraction ratio of 1 mg/mL overnight. For the PM scaffold condition, the scaffold was first placed on the coverslip before cell seeding. The seeding density was 2 × 10^4^ cells for both experimental groups. Cells were maintained in DMEM (Logan, UT, USA) supplemented with the corresponding HAp extraction medium under standard culture conditions (37 °C, 5% CO_2_, humidified atmosphere) for 3 days. Subsequently, the samples were fixed with 4% paraformaldehyde and permeabilized using 0.3% Triton X-100 purchased from Sigma Aldrich (St. Louis, MO, USA). F-actin was visualized using ReadyProbes™ phalloidin (Invitrogen, Waltham, MA, USA), while cell nuclei were counterstained with DAPI (Santa Cruz Biotechnology, Dallas, TX, USA). Fluorescence images were captured using a confocal laser scanning microscope (LSM 880, Carl Zeiss, Oberkochen, Germany).

#### Immunofluorescence Staining

Immunofluorescence staining was performed to evaluate angiogenesis-related marker VEGF expression. The experimental groups were limited to the HAp and HAp-CNF PM groups, and 2 × 10^4^ cells were seeded per well for both groups. For the HAp-CNF PM group, scaffolds were wrapped onto coverslips and placed into 48-well culture plates prior to cell seeding. For the HAp group, cells were also seeded on coverslips. HAp extracts were prepared in accordance with ISO 10993-12:2021 as above. Cells were cultured using the prepared extraction media where applicable. After 3 days of culture, the cells were fixed with 4% paraformaldehyde and subsequently permeabilized with 0.3% Triton X-100. Next, cells were incubated with 1% bovine serum albumin (BSA) for half an hour before overnight incubation with an anti-VEGF (1:200; sc-7269, Santa Cruz Biotechnology, TX, USA) primary antibody. After washing, this was followed by secondary anti-Mouse IgG Alexa Fluor™ 488 antibody (1:1000; A21202, Invitrogen, MA, USA) incubation and DAPI nuclei counterstaining. Fluorescence images were acquired using a confocal laser scanning microscope (LSM 880, Carl Zeiss, Oberkochen, Germany). Quantitative analysis of fluorescence intensity was performed using ImageJ software (Ver. 1.54), and mean fluorescence intensity (MFI) values were calculated to compare relative expression levels between the two groups.

### 2.6. Cell Viability Assay

Human bone marrow–derived mesenchymal stem cells (hBMSCs; PCS-500-012, ATCC, Manassas, VA, USA) were cultured in Dulbecco’s Modified Eagle Medium (DMEM) supplemented with 20% fetal bovine serum (FBS) and 1% penicillin/streptomycin. Cells were maintained under standard culture conditions (37 °C, 5% CO_2_, humidified atmosphere), with the culture medium replaced every two days. Extracts of HAp were prepared according to ISO 10993-12:2021 [29]. Briefly, the materials were immersed in complete culture medium at a concentration of 1 mg/mL and incubated overnight to generate extraction media. The PMs were cut into 1.2 cm × 1.2 cm specimens and sterilized by ultraviolet (UV) irradiation for 24 h. To maintain intimate contact between the matrices and the culture plate during cell culture, a sterilized rubber O-ring was positioned on top of each specimen in a 48-well plate (SPL Life Sciences, Pocheon-si, Republic of Korea). hBMSCs were then seeded onto the matrices at a density of 2 × 10^4^ cells per well in 1 mL of complete culture medium for all experimental groups. After 3 days of incubation, cell viability was assessed using a Cell Counting Kit-8 (CCK-8; Dojindo Laboratories, Kumamoto, Japan). Prior to the assay, the specimens were carefully washed with phosphate-buffered saline (PBS) to eliminate unattached cells. A working solution consisting of culture medium and CCK-8 reagent (10:1, *v*/*v*) was subsequently added to each well and incubated for 2 h at 37 °C. The CCK-8 assay is based on the conversion of the water-soluble tetrazolium salt WST-8 into a water-soluble formazan dye by cellular dehydrogenases in viable cells. The absorbance was measured at 450 nm using a microplate reader (SpectraMax, Molecular Devices, San Jose, CA, USA). Cell viability was determined from the optical density values and expressed as relative metabolic activity.

### 2.7. Alkaline Phosphatase (Alp) Activity Assay

hBMSCs were seeded into 48-well plates at a density of 2 × 10^4^ cells per well for all experimental groups and cultured under osteogenic conditions for 10 days. PM scaffolds were used as the cell culture substrate, and HAp extraction medium (1 mg/mL) was added to the osteogenic culture medium as required. HAp extraction medium was prepared in accordance with ISO 10993-12:2021 by incubating HAp in culture medium at an extraction ratio of 1 mg/mL overnight. After 10 days of osteogenic induction, ALP activity was evaluated using a TRACP & ALP Assay Kit (MK301, Takara Bio Inc., Shiga, Japan) according to the manufacturer’s instructions with slight modifications. The culture medium was removed, and the cells were washed once with physiological saline (Daihan Pharm Co., Ltd., Ansan, Republic of Korea). Cell lysis was performed by adding 150 μL of extraction buffer (physiological saline containing 1% NP-40) to each well, followed by gentle pipetting. Subsequently, 150 μL of substrate solution containing p-nitrophenyl phosphate (pNPP) prepared in ALP reaction buffer (0.2 M Tris-HCl, pH 9.5, 1 mM MgCl_2_) was added, and the reaction mixture was incubated at 37 °C for 1 h in a humidified CO_2_ incubator. The reaction was terminated by adding 150 μL of 0.5 N NaOH prepared from sodium hydroxide pellets (Sigma-Aldrich, St. Louis, MO, USA) dissolved in distilled water. Absorbance was measured at 405 nm using a microplate reader, and ALP activity was quantified from the optical density values.

### 2.8. Femur Defect Animal Model

Eight-week-old Sprague–Dawley rats were purchased from Koatech (Pyeongtaek, Republic of Korea). All animal experiments were conducted in accordance with institutional guidelines and were approved by the Institutional Animal Care and Use Committee (IACUC) of CHA University (Approval No. IACUC250138, Date: 25 August 2025). After acclimatization for one week, surgery was performed. A mixture of Rompun^®^ (10 mg/kg, Bayer Korea Ltd., Seoul, Republic of Korea) and Zoletil (0.4 mL/kg, Virbac Laboratories, Carros, France) was introduced via intraperitoneal administration for general anesthesia. To create a 2 mm defect, the femoral shaft was first exposed through a longitudinal skin incision, and a standard cylindrical defect was then created using a sterile 2-mm drill bit. The HAp scaffold was fabricated as a cylindrical construct measuring 2 mm in diameter and 1 mm in thickness and was implanted into the femoral defect measuring 2 mm in diameter and 2 mm in depth. The CNF PM was integrated with the HAp scaffold prior to implantation. The scaffold was positioned stably within the defect without the use of additional fixation materials such as adhesives or sutures. Following implantation, the surrounding tissues and surgical site were closed to maintain the implanted construct in position using a Visistat^®^ Skin Stapler (Teleflex, Wayne, PA, USA).

### 2.9. Histological Assessment and Immunohistochemical Analysis

Following specimen harvest, femoral tissues were fixed in 4% paraformaldehyde at room temperature and decalcified in 17% (*w*/*v*) EDTA solution (pH 7.4) for 28 days under gentle agitation. After complete decalcification, the samples were processed by sequential dehydration in graded ethanol, clearing with xylene, paraffin embedding, and sectioning into 7-μm-thick slices using a rotary microtome. Histological morphology was evaluated using hematoxylin and eosin (H&E) staining. Paraffin sections were deparaffinized with xylene and rehydrated through a graded ethanol series to distilled water. The sections were stained with hematoxylin to visualize cell nuclei, differentiated when required, blued in an alkaline solution, and subsequently counterstained with eosin. Following staining, the sections were dehydrated, cleared in xylene, and mounted using a permanent mounting medium. Immunohistochemical staining was performed to evaluate the expression of the osteogenic marker Osterix. Tissue sections were deparaffinized, rehydrated, and subjected to heat-induced antigen retrieval in citrate buffer (pH 6.0). Endogenous peroxidase activity was quenched with hydrogen peroxide, followed by blocking of nonspecific protein binding before primary antibody incubation. Sections were incubated overnight at 4 °C with primary antibodies against osterix (ab22552, Abcam), diluted 1:100. After washing, HRP-conjugated secondary antibodies were applied, and immunoreactivity was visualized using a DAB chromogen substrate kit (ab64261, Abcam) according to the manufacturer’s recommendations. Finally, the sections were counterstained with hematoxylin, dehydrated through graded ethanol, cleared in xylene, and mounted. Whole-slide images were acquired using a MoticEasyScan digital slide scanner (Motic Scientific, Universal City, TX, USA) and used for subsequent qualitative and quantitative analyses.

## 3. Results and Discussion

### 3.1. Fabrication and Structural Characterization of a Periosteum-Inspired Composite Scaffold

**Figure 1:**
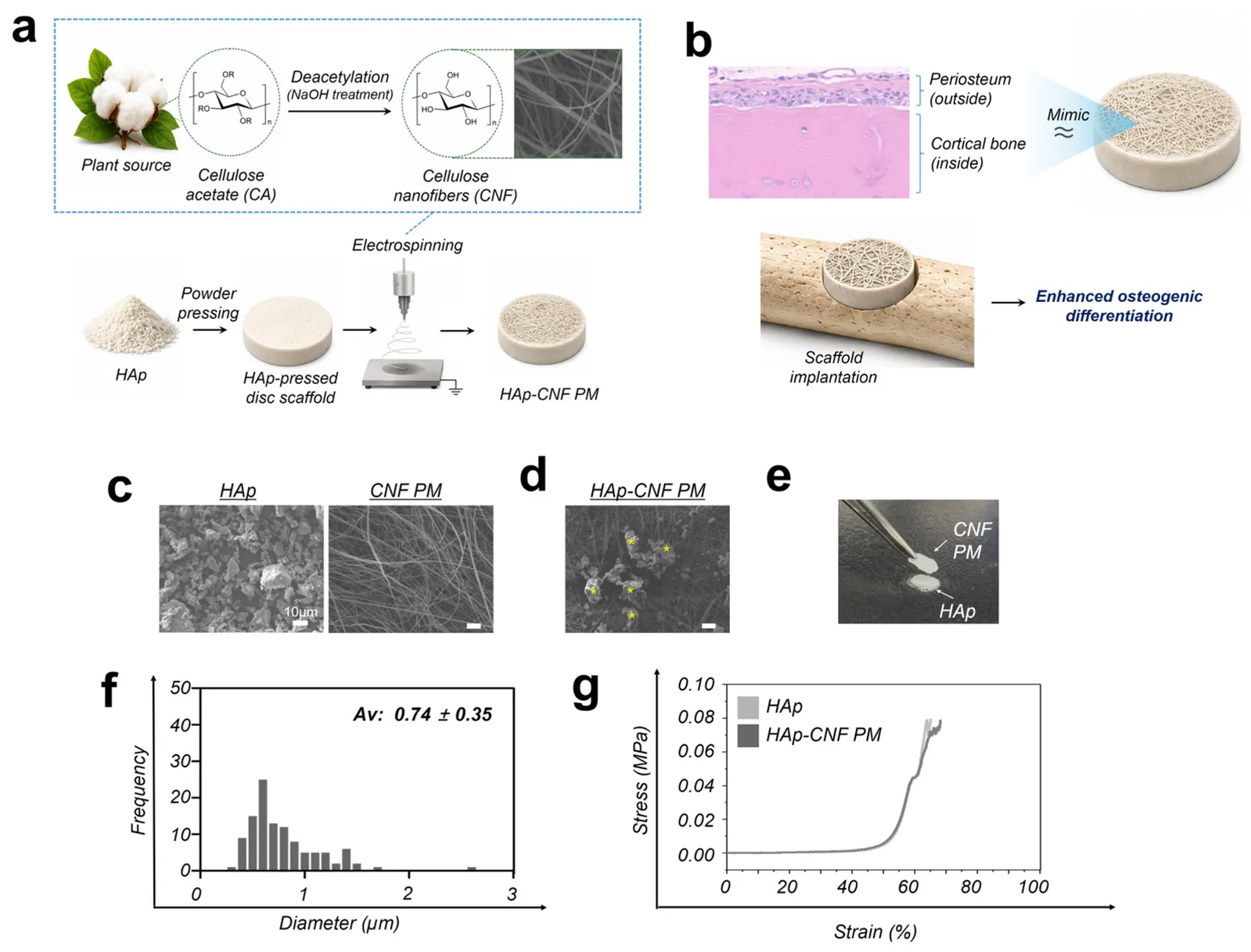
Fabrication and structural characterization of the periosteum-inspired HAp/CEL composite scaffold. Schematic illustration showing the fabrication process of the periosteum-inspired composite scaffold, in which electrospun CA nanofibers were converted into regenerated CEL through NaOH-mediated deacetylation to generate a CEL nanofibrous periosteum-mimetic membrane (CNF PM), followed by integration with a compressed HAp scaffold to reconstruct the periosteum–bone interface (**a**). Representative H&E image of native rat femoral tissue demonstrating the layered organization of cortical bone and periosteum (**b**). Scanning electron microscopy (SEM) images of the compressed HAp scaffold and the regenerated CEL nanofibrous membrane prior to assembly (**c**), and the assembled HAp-CNF PM composite scaffold after membrane integration (**d**). Representative digital photographs of the fabricated scaffolds, “*” represents HAp powder on CNF PM nanofibers (**e**), together with the corresponding fiber diameter distribution of the regenerated CEL nanofibrous membrane (**f**). Stress–strain graphs of HAp and HAp-CNF PM scaffolds (**g**).

To recapitulate the hierarchical architecture of native cortical bone and periosteum, a biomimetic composite scaffold was developed by integrating a regenerated CEL nanofibrous membrane with a compressed HAp scaffold (Figure 1a). CA, a plant-derived polysaccharide, was first electrospun into a nanofibrous membrane and subsequently converted into regenerated CEL through alkaline deacetylation using sodium hydroxide (NaOH). This deacetylation process generated a hydrophilic CEL nanofibrous membrane (CNF PM) while preserving the interconnected fibrous architecture, thereby providing a structural analogue of the native periosteal fibrous layer [28].

To reconstruct the mineralized component of cortical bone, HAp powder was mechanically compressed into cylindrical scaffolds (2 mm in diameter and 1 mm in thickness). The regenerated CEL membrane was then conformally integrated onto the HAp scaffold to produce a bilayer periosteum-inspired construct (HAp-CNF PM), consisting of a nanofibrous biological interface and a mechanically stable mineralized substrate.

Histological examination of native rat femoral tissue demonstrated the distinct layered organization of cortical bone covered by the periosteum (Figure 1b). This structural organization closely resembled the engineered composite scaffold, supporting the biomimetic design strategy in which the CEL nanofibrous membrane mimics the periosteal fibrous layer while the HAp scaffold reproduces the mineralized cortical bone [17,18].

SEM further confirmed the successful fabrication of each component (Figure 1c,d). The compressed HAp scaffold exhibited a densely packed particulate morphology composed of interconnected HAp particles, whereas the regenerated CEL membrane displayed a porous three-dimensional network of randomly oriented nanofibers. Following assembly, the CEL membrane was uniformly anchored onto the surface of the HAp scaffold while maintaining its fibrous architecture, resulting in an integrated bilayer structure that closely resembles the native periosteum–bone interface.

Macroscopic observation demonstrated that the CEL membrane uniformly covered the upper surface of the cylindrical HAp scaffold without noticeable delamination (Figure 1e), indicating stable integration between the fibrous membrane and the ceramic substrate. Quantitative analysis of fiber morphology revealed that the regenerated CEL membrane possessed an average fiber diameter of 0.74 ± 0.35 μm (Figure 1f), which falls within the microscale fibrous dimension reported for native periosteal collagen bundles and provides a highly porous extracellular matrix-like environment for cell attachment [19,22].

Investigation of the compressive mechanical properties of the HAp and HAp-CNF PM scaffolds provided further insight into their structural characteristics. Compressive testing was performed in triplicate (*n* = 3) for each group, and the resulting stress–strain profiles showed no substantial differences between the HAp and HAp-CNF PM scaffolds (Figure 1g). Although incorporation of the CEL membrane was intended to provide structural support to the HAp component, its presence did not result in a marked improvement in the overall compressive mechanical response under the conditions tested. These findings suggest that the CEL membrane primarily serves as a supporting and integrating component within the composite scaffold rather than substantially enhancing its bulk compressive mechanical properties.

### 3.2. Chemical and Crystalline Characterization of the Regenerated Cel Nanofibrous Membrane

**Figure 2:**
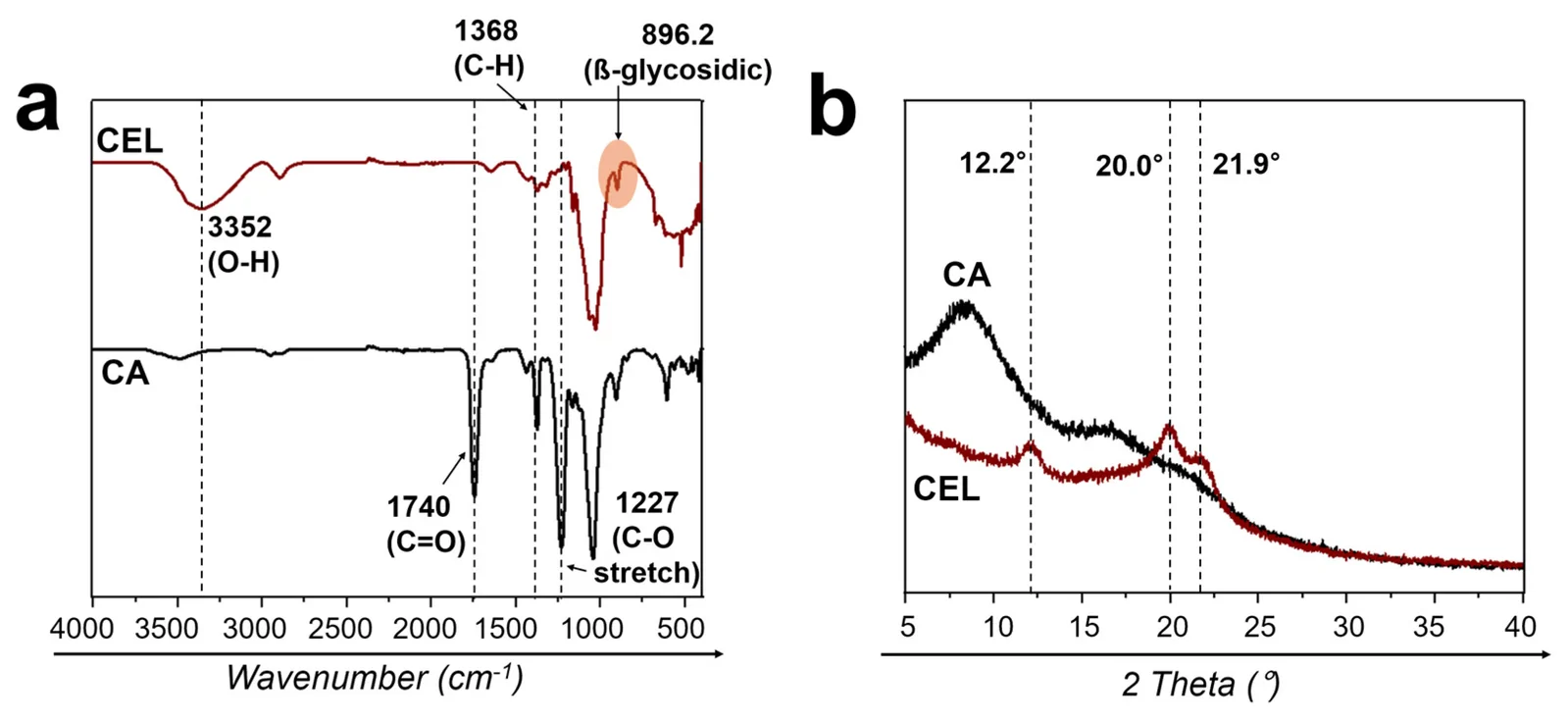
Chemical and crystalline characterization of the regenerated CEL nanofibrous membrane. Fourier-transform infrared (FT-IR) spectra of CA and regenerated CEL before and after NaOH-mediated deacetylation, demonstrating the disappearance of the characteristic ester carbonyl absorption of CA together with the appearance of characteristic CEL-associated absorption bands following regeneration (**a**). X-ray diffraction (XRD) patterns of CA and CEL showing the structural transformation induced by alkaline deacetylation and the emergence of characteristic diffraction reflections in the regenerated CEL membrane (**b**).

The successful conversion of CA into regenerated CEL was verified by FT-IR and XRD analyses (Figure 2a,b). As shown in the FT-IR spectra (Figure 2a), the regenerated CEL membrane exhibited a broad absorption band centered at 3352 cm^−1^, corresponding to the stretching vibration of hydrogen-bonded hydroxyl (O–H) groups [28]. The increased intensity of this band compared with CA indicates the regeneration of abundant hydroxyl groups following alkaline deacetylation. An absorption band at approximately 2900 cm^−1^ was assigned to the C–H stretching vibration of the glucopyranose backbone. The characteristic ester carbonyl (C=O) stretching band of CA at approximately 1735 cm^−1^ was markedly diminished after NaOH treatment, confirming the effective removal of acetyl groups during the deacetylation process. The absorption band at 1023 cm^−1^, corresponding to C–O stretching vibrations of the CEL backbone, was clearly retained after deacetylation [28]. In addition, the appearance of a characteristic absorption band at approximately 897 cm^−1^, associated with the β-(1→4)-glycosidic linkage of CEL, provided additional evidence for the successful conversion of CA into regenerated CEL.

The structural transformation following alkaline deacetylation was further confirmed by XRD analysis (Figure 2b). In contrast to CA, which exhibited a broad diffraction pattern characteristic of its relatively low crystallinity, the regenerated CEL membrane displayed characteristic diffraction reflections at 2θ = 12.2°, 20.0°, and 21.9°, consistent with the formation of regenerated CEL after alkaline treatment [10,28]. The appearance of these reflections indicates molecular rearrangement of the CEL chains during the regeneration process. Together with the FT-IR and SEM analyses, these findings confirm the successful conversion of electrospun CA into regenerated CEL while preserving the nanofibrous architecture.

### 3.3. Cellular Compatibility and Early Osteogenic Response of the Periosteum-Inspired Composite Scaffold

**Figure 3:**
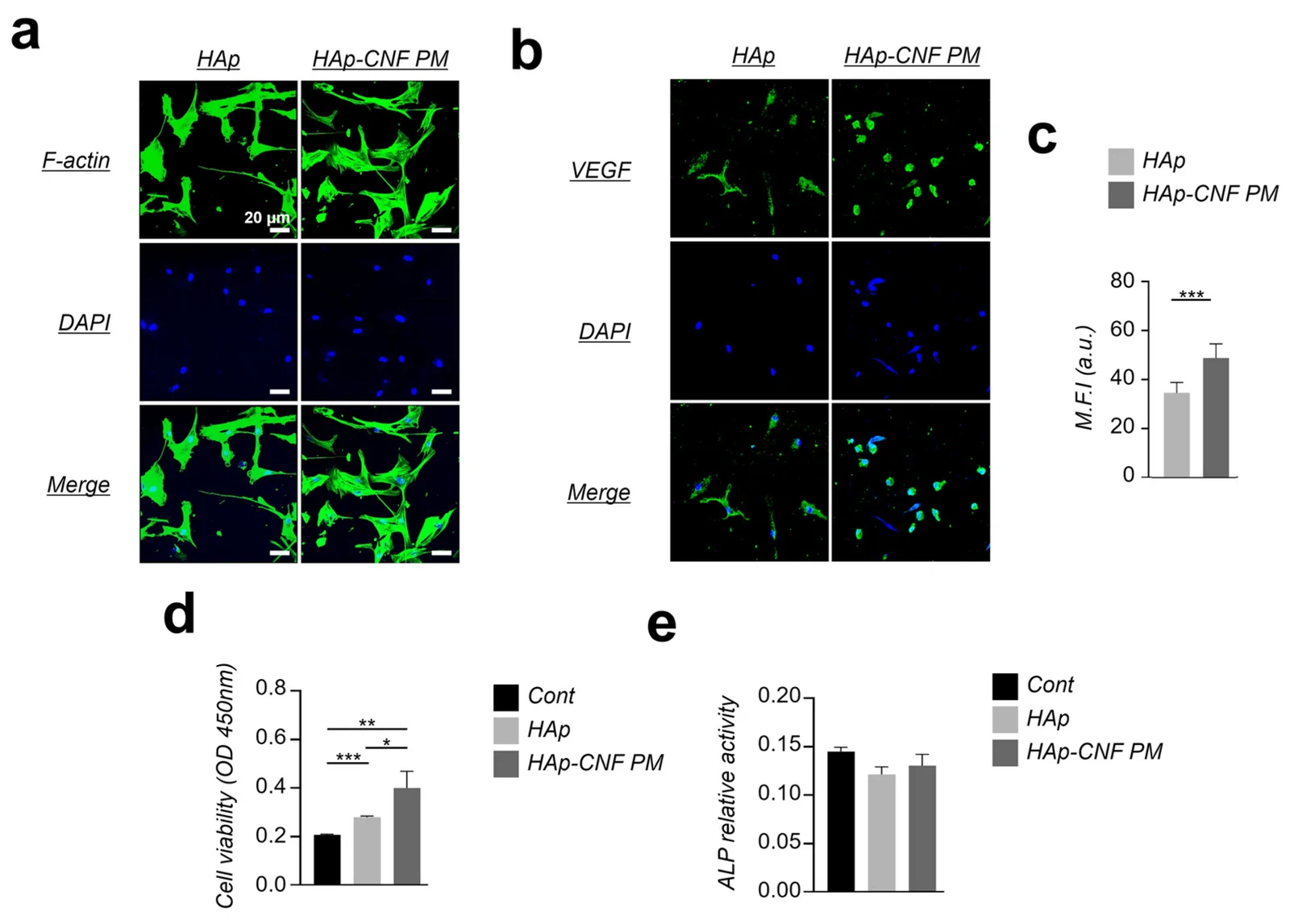
In vitro cellular responses to the periosteum-inspired HAp-CNF PM composite scaffold. Representative fluorescence images of cells cultured on the tissue culture plate control, compressed HAp scaffold, and HAp-CNF PM composite scaffold, showing F-actin cytoskeletons and DAPI-stained nuclei (**a**). Confocal images (**b**) and quantification (**c**) of VEGF expression on cells seeded on HAp and HAp-CNF PM scaffolds. Quantitative evaluation of cellular metabolic activity using the CCK-8 assay (**d**) and alkaline phosphatase activity as an indicator of the early osteogenic response (**e**). Data are presented as mean ± SD. Statistical significance: * *p* < 0.05, ** *p* < 0.01, *** *p* < 0.001.

The cytocompatibility of the periosteum-inspired composite scaffold was evaluated using cytoskeletal fluorescence imaging, CCK-8 analysis, and alkaline phosphatase (ALP) activity assays. Fluorescence staining of F-actin and nuclei revealed well-spread cells with clearly organized actin filaments in the HAp and HAp-CNF PM groups (Figure 3a). No marked differences in overall cellular morphology were observed among the groups, indicating that neither the compressed HAp scaffold nor the CEL nanofibrous membrane adversely affected cell attachment or cytoskeletal organization under the tested culture conditions.

Immunofluorescence staining of the angiogenesis-related marker VEGF on HAp and HAp-CNF PM scaffolds was carried out to further evaluate whether the periosteum-inspired composite scaffold supports vascular responses during bone regeneration. Expression of VEGF in the HAp-CNF PM group was significantly higher than in the HAp group, as shown in Figure 3b. This was confirmed by quantification results in Figure 3c, suggesting that the CEL nanofibrous membrane provides more favorable conditions which support the regenerative microenvironment.

Quantitative CCK-8 analysis demonstrated significantly greater cellular metabolic activity in the HAp-containing groups than in the tissue culture plate control (Figure 3d). Moreover, integration of the CNF PM with the HAp scaffold resulted in a further increase in the CCK-8 signal, with the HAp-CNF PM group exhibiting the highest value among the tested conditions. Electrospun CEL-based fibrous matrices provide a highly interconnected architecture and a large surface area available for cell–material interactions, while regenerated CEL contains abundant surface hydroxyl groups that support hydration and adsorption of cell-interacting proteins [27,28]. These physicochemical and topographical characteristics may contribute to the favorable cytocompatibility observed for the CNF PM-containing scaffold. Previous studies have similarly demonstrated that electrospun regenerated CEL and CA-based fibrous scaffolds can support osteoblast adhesion, spreading, and proliferation [27,28].

ALP activity was subsequently assessed to examine the early osteogenic response to the scaffolds (Figure 3e). The HAp-containing groups exhibited slightly lower ALP activity than the tissue culture plate control, although incorporation of the CNF PM partially increased the activity relative to the HAp-only group. This result indicates that the enhanced CCK-8 response of the HAp-CNF PM scaffold did not translate into a proportionate increase in ALP activity under the present experimental conditions. Because cellular proliferation and osteogenic differentiation are not necessarily coupled, the higher metabolic activity of the composite scaffold should be interpreted independently from its early osteogenic response [30].

The relatively low ALP activity measured in the presence of HAp may have also been influenced, at least in part, by scaffold-associated recovery or assay effects [31,32]. Calcium phosphate ceramics, including HAp, exhibit substantial protein adsorption through interactions involving exposed calcium and phosphate sites, a property that has enabled their extensive use as chromatographic matrices for protein separation [32]. Accordingly, adsorption of ALP-containing cellular proteins onto the ceramic surface or incomplete recovery of lysates from the porous scaffold cannot be excluded.

Although the CEL nanofibrous membrane provides a favorable interface for cell attachment and metabolic activity, its coverage of the HAp surface may partially limit direct cell–HAp interactions and reduce cellular exposure to calcium and phosphate ions released from or present at the mineral surface. Such reduced interaction with the mineral phase may attenuate HAp-mediated osteogenic stimulation, potentially explaining why the increase in CCK-8 activity was not accompanied by a proportional increase in ALP activity.

These findings demonstrate that coupling the regenerated CEL nanofibrous membrane to the compressed HAp substrate enhanced cellular compatibility while maintaining ALP activity at a level higher than that observed for the HAp scaffold alone. Thus, the CNF PM served primarily as a cell-supportive fibrous interface rather than as a strong independent inducer of early osteogenic differentiation.

### 3.4. In Vivo Bone Regeneration Promoted by the Periosteum-Inspired Composite Scaffold

**Figure 4:**
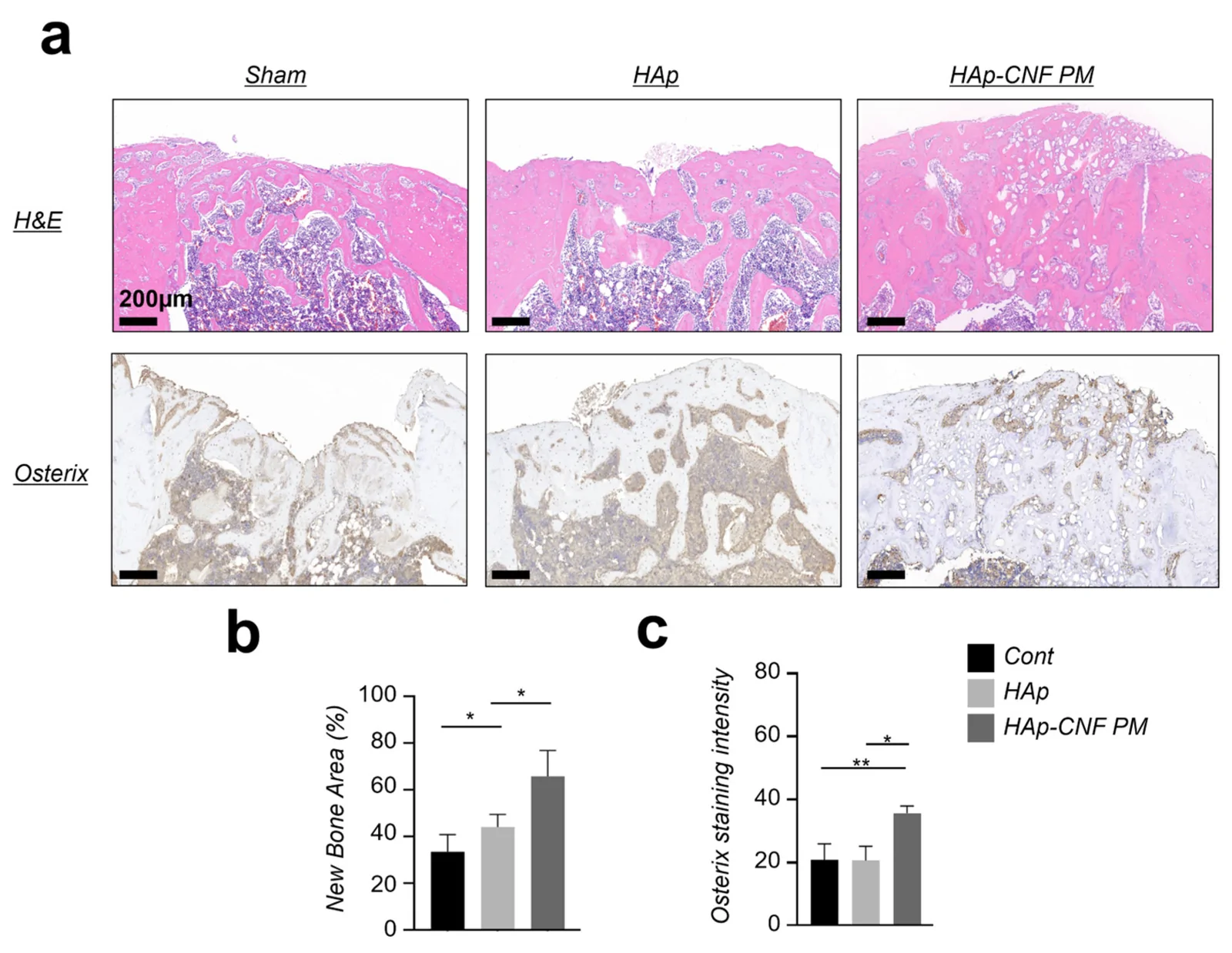
In vivo evaluation of bone regeneration following implantation of the periosteum-inspired composite scaffold in a rat femoral defect model. Representative H&E and Osterix immunohistochemical staining images of defect tissues harvested from the sham, HAp, and HAp-CNFPM groups following implantation into a cylindrical femoral cortical defect (2 mm × 2 mm) (**a**). Quantitative analysis of newly formed bone area based on H&E staining (**b**) and Osterix-positive area determined by immunohistochemistry (**c**). Data are presented as mean ± SD. Statistical significance: * *p* < 0.05, ** *p* < 0.01.

To evaluate the bone regenerative capacity of the periosteum-inspired composite scaffold, cylindrical scaffolds were implanted into a critical-sized femoral cortical defect (2 mm in diameter × 2 mm in depth) in SD rats (Figure 4a). For the in vivo study, both 1- and 3-week time points were initially considered during the experimental design. However, during preliminary evaluation of the 1-week specimens, the implanted mineral material remained locally aggregated within the defect, with limited tissue ingrowth into and around the mineral aggregates. Under these early conditions, the retained mineral aggregates could not be reliably distinguished from tissue-associated regenerative changes for quantitative assessment, potentially leading to misinterpretation of the early regenerative response. Therefore, the 1-week specimens were not included in the quantitative evaluation of bone regeneration, and 3 weeks was selected as the primary endpoint, at which tissue integration and newly formed bone could be more reliably evaluated histologically.

Histological evaluation using H&E staining revealed limited spontaneous bone regeneration in the sham group, with the defect area remaining largely occupied by fibrous tissue. In contrast, implantation of the HAp scaffold resulted in appreciable new bone formation extending from the defect margins toward the implanted scaffold. The HAp-CNF PM group exhibited substantially greater new bone formation, with newly formed bone occupying a larger proportion of the defect area and showing more continuous integration with the surrounding host bone.

Quantitative analysis of the newly formed bone area further supported the histological observations (Figure 4b). The HAp scaffold significantly increased new bone formation compared with the sham group, whereas incorporation of the CEL nanofibrous periosteum-mimetic membrane produced an additional and significant enhancement in bone regeneration. These findings suggest that incorporation of the regenerated CEL membrane into the HAp scaffold provides a biologically favorable interface associated with enhanced tissue integration and bone regeneration compared with the HAp scaffold alone. The nanofibrous architecture of regenerated CEL closely resembles the fibrous extracellular matrix of the native periosteum, thereby providing an environment that supports cell infiltration and host tissue interaction, ultimately contributing to enhanced bone regeneration [10,27,28].

Immunohistochemical staining for Osterix was subsequently performed to evaluate osteogenic differentiation within the regenerated tissue (Figure 4a). While the HAp scaffold alone did not significantly increase Osterix-positive cells compared with the sham group, the HAp-CNF PM scaffold exhibited a marked increase in Osterix expression throughout the newly regenerated tissue. Quantitative analysis confirmed a significant increase in Osterix-positive area in the HAp-CNF PM group compared with both the sham and HAp groups (Figure 4c). Because Osterix is an essential transcription factor governing osteoblast differentiation and bone matrix formation, these findings indicate that incorporation of the periosteum-mimetic CEL membrane promoted osteogenic commitment within the defect region beyond that achieved by the mineralized HAp scaffold alone.

These in vivo results demonstrate that, although the compressed HAp scaffold provided an osteoconductive mineral framework, incorporation of the regenerated CEL nanofibrous membrane was associated with enhanced new bone formation and osteogenic differentiation. These findings highlight the potential benefit of combining a mineralized ceramic framework with a fibrous periosteum-mimetic interface for bone regeneration.

Overall, regenerated CEL has numerous advantages including its simple fabrication process, cost-effectiveness, hydrophilicity, and extracellular matrix-like fibrous architecture. In particular, the abundant hydroxyl groups of regenerated CEL provide a hydrophilic surface favorable for cell–material interactions, while the electrospinning and subsequent deacetylation process enables fabrication of a periosteum-mimetic fibrous membrane without requiring complex functionalization. Furthermore, our scaffold is entirely acellular and does not require exogenous growth factors or other bioactive molecules, which may reduce fabrication complexity and facilitate scalability and translational applicability. At the same time, we acknowledge that this simplicity also represents a limitation of the present system. The CEL membrane primarily reproduces the structural and cell-supportive characteristics of the periosteal interface and does not fully recapitulate the complex biological functions of native periosteum, particularly those mediated by osteogenic or angiogenic bioactive molecules. Therefore, the incorporation of appropriate bioactive cues may be considered in future studies to further enhance the regenerative functionality of the scaffold.

## 4. Conclusions

In this study, we developed a periosteum-inspired composite scaffold by integrating a regenerated CEL nanofibrous membrane onto a compressed HAp scaffold to reconstruct both the biological interface and the mineralized framework of native bone. The regenerated CEL membrane, fabricated through NaOH-mediated deacetylation of electrospun CA, preserved its extracellular matrix-like fibrous architecture while providing a favorable surface for cell–material interactions. Although the HAp scaffold alone primarily served as a mechanically stable osteoconductive substrate, incorporation of the periosteum-mimetic membrane positively influenced cellular compatibility and promoted in vivo bone regeneration. Histological analyses demonstrated substantially greater new bone formation and elevated Osterix expression in defects treated with the HAp-CNF PM scaffold compared with the HAp scaffold alone, indicating enhanced osteogenic commitment during bone repair. These findings highlight that reconstructing the periosteal microenvironment, rather than relying solely on a mineralized scaffold, represents an effective strategy for improving endogenous bone regeneration. The proposed biomimetic design provides a simple and acellular platform with potential for application as a bone graft substitute. However, several limitations of this study should be acknowledged, including the lack of a CEL-membrane-only control, the limited evaluation time points, and the absence of long-term assessment of CEL membrane persistence and remodeling. Regenerated cellulose has limited biodegradability in vivo because mammalian tissues lack cellulolytic enzymes capable of efficiently degrading cellulose [33,34,35]. Consequently, the CEL membrane may persist at the implantation site for an extended period, and its long-term fate was not evaluated in the present study. Further studies are therefore required to determine membrane persistence and structural changes, tissue integration or possible fibrous encapsulation, associated host responses, and potential effects on long-term bone remodeling. Such long-term in vivo evaluations will be important for establishing the safety and translational applicability of the proposed scaffold.

### Statistical Analysis

Data are presented as mean ± standard error of the mean (SEM). For comparisons between two groups, statistical significance was evaluated using an unpaired two-tailed Student’s *t*-test. For multiple group comparisons (≥3 groups), one-way ANOVA followed by Tukey’s post hoc test was applied. A *p*-value < 0.05 was considered statistically significant. Significance levels were denoted as * *p* < 0.05, ** *p* < 0.01, and *** *p* < 0.001 versus the control group, unless otherwise specified.

## Data Availability

The original contributions presented in the study are included in the article; further inquiries can be directed to the corresponding authors.
